# Pain in Multiple Sclerosis: Understanding Pathophysiology, Diagnosis, and Management Through Clinical Vignettes

**DOI:** 10.3389/fneur.2021.799698

**Published:** 2022-01-13

**Authors:** Michael K. Racke, Elliot M. Frohman, Teresa Frohman

**Affiliations:** ^1^Department of Medical Affairs, Quest Diagnostics, Secaucus, NJ, United States; ^2^Neuroimmunology Laboratory of Lawrence Steinman, Stanford University School of Medicine, Stanford, CA, United States

**Keywords:** multiple sclerosis, pain, pathophysiology, treatment, diagnosis

## Abstract

Neuropathic pain and other pain syndromes occur in the vast majority of patients with multiple sclerosis at some time during their disease course. Pain can become chronic and paroxysmal. In this review, we will utilize clinical vignettes to describe various pain syndromes associated with multiple sclerosis and their pathophysiology. These syndromes vary from central neuropathic pain or Lhermitte's phenomenon associated with central nervous system lesions to trigeminal neuralgia and optic neuritis pain associated with nerve lesions. Muscular pain can also arise due to spasticity. In addition, we will discuss strategies utilized to help patients manage these symptoms.

## Introduction

Multiple sclerosis (MS) is an inflammatory, demyelinating disorder of the central nervous system (CNS). Lesions commonly occur in the white matter of the brain and spinal cord, and can also occur in the brainstem and optic nerves (1). Not surprisingly, pain is a common occurrence in multiple sclerosis. Pain can occur as an acute syndrome, but chronic pain occurs in 50–75% of MS patients at some time during their disease course (2). Pain can be the initial manifestation of MS and this occurs in about 10% of MS patients. The type of pain syndrome that occurs in MS is typically associated with the part of the nervous system that is involved but has little to do with the type of MS or for how long the patient has had the disease (3).

In this review, we will utilize case vignettes to help describe scenarios where pain commonly occurs in MS and describe the associated pathophysiology. The clinical vignette approach allows one to examine the management of a complex case, which is often the situation in MS patients experiencing significant pain. Patients with MS present with a wide variety of symptoms, from central neuropathic pain to Lhermitte's sign to dysesthesias (see Table 1). This variable presentation also holds true for pain that results from the various types of CNS involvement in MS. This review emphasizes that it is important to characterize the type of pain properly in order to treat patients appropriately. Many patients with MS have reported poor outcomes because their physicians failed to categorize and treat their pain adequately. We will also describe strategies to help clinicians manage situations in which MS patients are experiencing pain, often based on the authors experience in clinical practice. We note that names in the clinical vignettes are fictitious, but they do reflect actual scenarios that clinicians may experience in their care of MS patients.

**Table 1:** Glossary of terms applicable to MS pain subtypes.

**Pain term**	**Categorical definition**
Allodynia	Provocation of unexpected pain in response to a non-painful stimulus
Analgesia	Absence of pain
Anesthesia Dolorosa	Pain in a region that is anesthetic; a form of “deafferentation pain”. The term literally means; “painful numbness”
Causalgia: “complex regional pain syndrome” (CRPS); “reflex sympathetic dystrophy”	Severe burning sensation, and exaggerated pain sensation in response to non-painful stimuli secondary to peripheral nerve injury, however CNS lesions, principally spinal cord lesions, can also produce CRPS; often associated with changes in skin color, nail and hair growth.
Central neuropathic pain	Pain from a CNS lesion, affecting somatosensory networks
Deafferentation pain	Pain occurring in a region that has become anesthetic secondary to a sensory syndrome resulting in the perception of anesthetic numbness, but which later transforms into a region of pain generated by a non-painful stimulus (e.g. anesthesia dolorosa)
Dysesthesia	An unpleasant sensation that can occur spontaneously or can be evoked
Dyspareunia	Pain with sexual intercourse
Hyperalgesia	An exaggerated pain response to a normally painful stimulus
Hyperesthesia	An exaggerated sensitivity associated with any of the five senses, sight, sound, taste, touch, or smell
Hyperpathia	An abnormally painful response reaction to a given stimulus
Hypoalgesia	An attenuated pain response to a normally painful stimulus
Lhermitte's sign	Electrical and/or vibratory sensation triggered by neck flexion, with the pattern of radiation typically moving downward into the neck, arms, and back. Rostral or ascending sensation from the neck into the back of the head (in a C2 distribution can also occur). A similar provoked sensation with movement can be likewise precipitated in a lumbo-sacral distribution following prolonged sitting and ascending quickly.
‘MS Hug'	An uncomfortable tonic squeezing or rhythmic oscillating muscular activation of thoracic, intercostal, or myotomal (motor root distribution) activation secondary to ephaptic discharges from the spinal cord
Neuralgia	Pain in the distribution of a nerve or multiple nerves
Neuritis pain	Pain secondary to inflammation of nerve(s)
Nociception	Neurological process by which painful/noxious stimuli are encoded and processed within pain networks
Paresthesias	An abnormal sensation that is not necessarily unpleasant but can be disturbing
Phantom pain	Pain that is perceived to be coming from a body part that is no longer present anatomically, but whose central representation persists, thereby capable of producing painful or abnormal symptoms that the brain perceives as localized to a body-part in anatomic space that is no longer present
Radicular pain	Pain with a pathway of radiation that follows a particular nerve root distribution; typically, secondary to mechanical factors such as disc herniation
Pseudoradicular pain	Pain that appears to radiate in a particular nerve root distribution, not caused by mechanical factors, but rather, occurs secondary to an MS plaque of demyelination localized to the very proximal sensory root, where myelin is provided by oligodendrocytes

## “Someone Plugged My Face into an Electric Powerplant”: Pain Associated With Trigeminal Neuralgia (TN)

Hope was a 32-year-old woman with a known history of relapsing remitting multiple sclerosis (RRMS) and no known other concomitant conditions. She presented to clinic complaining of severe paroxysmal pain in the right VI and V2 region of the trigeminal nerve that occurred first after her husband gently caressed her right cheek.

The pain was described as “sharp, stabbing and like an electric shock”; “actually it was like someone plugged my face into an electric powerplant.” The pain lasted just seconds but could be reproduced by lightly rubbing her face with a towel after washing, by wind blowing on it, by brushing her teeth and by eating. It was absent between these events and could not be reproduced by roughly “poking and prodding” the affected area. The patient indicated that the pain was so severe that each episode provoked her to tears. Further, she has lost 10 pounds in the last month because the pain was triggered while eating and drinking. She relates that she does not experience a predictable period of reprieve at any time during the day or night.

Hope's neurological exam was unchanged compared to her clinic visit 3 months prior, with the exception of a subtle hyperesthesia localized to the right face in a V1/V2 distribution, directly superimposed upon the distribution of her pain. A recent trip to the dentist for the pain did not elucidate a dental issue.

A MRI (magnetic resonance imaging) was obtained that was similar to images taken 6 months earlier and showed demyelinating brainstem lesions in the left paramedian pontine tegmentum. In addition, the MRI showed a new enhancing plaque localized to the right dorsal midbrain/pons in the area of the trigeminal pathways. No other lesions or masses were identified. Likewise, MR angiography and MR cisternogram failed to reveal evidence of a vascular ectasia in the region of the trigeminal root system.

Given the presence of a newly enhancing lesion, Hope was treated with a 5-day course of intravenous methylprednisolone (IVMP) at 1 gm daily. As with past exacerbations, she exhibited the most robust, fast-acting, and improvement capability using IVMP when compared to oral steroid regimens of commensurate dosing.

While she markedly improved with respect to the magnitude of the pain's intensity and frequency, Hope continued to experience multiple and debilitating shocks in the right face throughout the day. A second course of IVMP at 1 gm daily for 3 days exerted no further benefit. Given the acuity in the emergence of this not uncommon MS-associated syndrome, it was decided to advance therapy to plasma exchange at 1 plasma volume exchange per day, for a total of five volume exchanges requiring a total of 7 days (given the need to allow autocorrection of fibrinogen and the desire to not use fresh frozen plasma). Despite this therapy, Hope remained unchanged, thereby suggesting that the new plaque, and its corresponding symptom complex was not wholly based upon inflammation, but that some trigeminal circuit reorganization had occurred in the context of inflammatory demyelination ultimately leading to so-called “ephaptic” discharges. With this mechanism in mind, Hope was started on a membrane-stabilizing agent, carbamazepine at 100 mg/day and titrated up to 400 mg/day twice daily, which was effective for pain relief, but the side effects were not tolerable. She was switched to oxycarbazepine at 150 mg twice daily and titrating every 3 days to a dose of 450 mg bid, at which point she achieved excellent and well-tolerated pain control (see Table 2).

**Table 2:** MS-associated pain syndromes and potential treatment strategies.

**Pain syndrome**	**Characteristics**	**Treatment strategy**
Deafferentation dysesthetic	Exaggerated sensation in a region perceived as numb or reduced in sensation	Membrane stabilizers GabapentinoidsTopical sprays Aripiprazole (personal obs)
Dysesthetic ‘itching'	Intense cutaneous itch in a dermatome, or set of them	Ethyl Chloride spray Lidocaine or benzocaine spray (sunburn spray) Cetacaine spray or gel **Chronic:** Membrane stabilizers Tricyclic anti-depressants Gabapentinoids Aripiprazole (personal obs)
Dyspareunia	During intercourse; often with colorful description (e.g. sliding ground glass, chains, hot or cold metal	Topical gabapentin (8-12%) applied at the vaginal introitus Belladonna and Opium supprettes (B&O) Oral agents
Dysuria	Pain with urination	Phenazopyridine generally dosed at 100-200mg taken up to 3x/day after meals Taken for up to 2 days if used in conjunction with anti-microbial agents for a UTI Taken chronically for chronic dysuria Caution: This agent can cause a reddish-orange color of the urine; and can stain fabrics
Focal dystonia	Typically hand/foot	Baclofen Benzodiazepines Tizanidine
Genu recurvatum	Knee pain from hyperextension of the knee during the stance phase of the leg at the end swing phase; due to unopposed action of the quadriceps and gluteus maximus	Dorsiflexion assist (e.g. with an ankle-foot-orthosis; AFO; either single piece or articulated if the patient has adequate control of the foot) of the foot will add ‘flexion moment' to the knee, reducing this chronic problem in many MS patients with gait dysfunction
Glossopharyngeal neuralgia Glossopharyngeal vagal neuralgia	Extreme pain in the throat, tonsillar region, and/or posterior tongue. Paroxysmal. Can radiate to the ear, and can occur with vagal activation leading to syncope	Topical spray for reducing pain crisis Oral Membrane stabilizers Vascular decompression surgery
Ice-Cold; Burning-Hot, Black and Blue, Toe, Foot, Ankle Neuropathic-Causalgic pain syndrome	Usually on the basis of dysautonomia, associated with color changes (acrocyanosis), temperature changes (usually cold feet), changes in hair and nail growth, and edema (secondary to weakness, venous valvular incompetence with age, and dysautonomia	Membrane stabilizers Citostazol: an anti-platelet and vasodilator agent that we have found to be the most effective agent for this problem. Starting at 25mg qhs and titrating weekly to 100-200mg daily to bid; always taken on an empty stomach; 30-60 min before, or 2 h after a meal
Indwelling catheter pain	Associated with chronic use of an indwelling or Foley catheter or a suprapubic catheter.	We generally suggest topical ‘triple antibiotic' as these have both anti-microbial and analgesic properties **Topicals:** Gabapentin (8-12% Lidocaine: (2-4%) Benzocaine: (10-20%) Cetacaine Gel:
Irrational exuberance of arousal & orgasm	Exaggerated arousal and orgasmic reflexes; whereby pleasure can transform into perception of overstimulation, spike-like and rapid sequential orgasms in women in contrast to a singular intense and longer than normal duration orgasm in men	Membrane Stabilizers Tricyclic anti-depressants Gabapentinoids **Topicals:** Gabapentin (8-12%) Gabapentin + Clonidine (5%) Lidocaine 2-4% gel Benzocaine 10-20% gel Cetacaine gel
Levator ani pain syndrome	Similar to proctalgia fugax (see below), although the pain is perceived to be localized to the pelvic floor or higher. Prolonged sitting can contribute to this syndrome.	Stretching Avoid prolonged sitting in the same position Muscle relaxers Baclofen Tizanidine Benzodiazepines
Lhermitte's	Typically in the context of a cervical MS plaque, with ephaptic discharges from the dorsal root entry zone (pseudoradicular from proximal root demyelination), Lissauer's tract, substantia gelatinosa, or spinothalamic tract.	Acute from a new MS plaque will generally respond to corticosteroids Chronic reemergent Lhermitte's when frequent, painful, and recalcitrant will respond to membrane stabilizers, gabapentanoids, pulse steroids, ethyl chloride spray
Lumbar puncture intracranial hypotension associated headache	From CSF leak; most common when using a Quincke cutting needle (and from a large gauge needle); very uncommon when using non-cutting pencil point needles such as Sprotte and Whitacre, at 20-22 gauge.	Prevention is the key, by using a non-cutting, pencil point needles such as the Sprotte and the Whitacre types at 20-22 guage. Blood patch is the most effective method to abort a CSF leak syndrome (headache when upright); whether from an LP or spontaneous leak from a breach in the integrity of a Tarlov nerve-root sleeve.
Migraine and tension headaches	More common in MS than in the general population.	Typical migraine abortive and prophylactic therapy.
MS Hug	An uncomfortable tonic squeezing or rhythmic oscillating muscular activation of thoracic, intercostal, or myotomal (motor root distribution) activation secondary to ephaptic discharges from the spinal cord	**Tonic:** Baclofen Tizanidine Dantrolene **Phasic:** Clonazepam Diazepam Levetiracetam
Occipital neuralgia	Occipital ridge pain, which can be triggered by palpation and pressure application	Tender zone injection with local anesthetic and methylprednisolone. BoTox Ethyl Chloride spray and massage
Proctalgia fugax	Exaggerated ephaptic innervation of the rectal sphincters leading to rectal pain and protracted sphincter activation, which can impede defecation. Similar to the Levator ani syndrome, the former involves pain localized to the rectum and sphincters, while the latter is localized to the pelvic floor or higher.	Membrane stabilizers B&O Supprettes **Topical gels:** Apply to rectum and anal region: Gabapentin (8-12%) Lidocaine (2-4%) Benzocaine (10-20%) Cetacaine Gel
Sacral cutaneous pain	From prolonged sitting, with or without sacral decubitus	Offload pressure by regular dynamic change in sitting posture and ‘attitude'. Tilt option on electric wheelchairs can serve to avoid pressure at a constant point of contact of the patient's sacrum with the sitting device.
Spasticity: Tonic Phasic Tendon shortening/Contraction pain	Exaggerated activity from unchecked (reduced central inhibition) to skeletal muscles	**Tonic:** Baclofen Tizanidine Dantrolene **Phasic:** Benzodiazepines Baclofen Levetiracetam Stretching and Exercise
Trigeminal neuralgia	Central ephaptic discharges along the trigeminal tract system. The pain can be along V1, V2, or V3 roots of the trigeminal nerve (V), but can also occur in an onion skin pattern affecting the central face (from damage to the pars oralis; most rostral part of the trigeminal tract); mid lateral face (affecting the pars interpolaris); or the extreme lateral face (affecting the pars caudalis); whereas the trigeminal tract descends as low as C4 and is contiguous with the substantial gelatinosa.	Membrane stabilizers Gabapentanoids Tricyclic anti-depressants Misoprostil Aripiprazole (personal obs) Vascular decompression Rhizotomy Gamma knife
Vaginismus +/- Penis captivus	Arousal mediated escalation in the motor response activation of the vaginal vault muscles, especially those which contribute to the introitus. The response is of sufficient magnitude in order to tonically and synchronously active the vaginal entry muscle apparatus,	**Pre-emptive: 30–60 min prior to sexual activity** Relaxation techniques Membrane Stabilizers Baclofen Benzodiazepines
	such that if it occurs during intercourse, the penis shall be entrapped or captured, and fully unable to exit the powerful force of the introitus circumferentially exerting a form of ‘ring compression' around the shaft of the penis; very much akin to an ‘O' ring.	**Topical: Applied to Introitus** Gabapentin gel (8–12%) Dextromethorphan (5–10%) Clonidiine (5–8%) Lidocaine (2–4%) Benzocaine (5–10%) Drink a can of tonic water containing quinine one hour prior to sexual activity.

While Hope remained in good control for several years without any identifiable antecedent, the pain returned. Several other agents were added, including lamotrigine and misoprostal. This three-drug regimen produced adequate control, albeit with refractory side effects most prominently sedation and poor concentration at work and at home.

Hope was then referred to Neurosurgery for an evaluation of interventions capable of mitigating, if not abolishing TNin those with vascular compression syndromes, but also in those with MS. Her surgeonemphasized the superior outcomes when a retroauricular, suboccipital, and extradural decompression procedure is performed in conjunction with a high precision cut of the involved sensory root(s); as the first surgical intervention, rather than utilizing rhizotomy or gamma knife (4).

Even when no vascular compression is identified intraoperatively, skipping the first step (vascular decompression with the placement of a pledget between the involved vessel and the affiliated trigeminal root) and advancing to the “cut” of the sensory root can be effective if not curative in those with MS-associated trigeminal neuralgia (5).

Hope agreed to proceed with the right sided trigeminal decompression procedure, where the lack of an ectatic vessel prompted the surgeon to limit the procedure to the second step, essentially involving the “cut” of the V2/V3 sensory root. From this procedure Hope experienced about an 80% reduction in both the frequency and intensity of her pain, while still requiring some medication to control the pain (i.e., with oxcarbazepine at 450 mg twice daily).

Upon follow up, the surgeon informed Hope that the residual pain could be eradicated by advancing to step 2 in her usual treatment sequence; specifically selective V2/V3 rhizotomies. Hope agreed and the procedure was a resounding success; even allowing her to taper completely off the oxcarbazepine and prompting her to say; “I feel like my old self before the trigeminal neuralgia began.” She remains pain free 7 years later.

TN is defined as sudden paroxysmal severe stabbing “electrical” pain that is brief in onset and cessation, and usually unilateral. It occurs in recurrent episodes and is limited to the distribution of one or more of the trigeminal nerve branches, typically triggered by innocuous sensory stimuli (6–8). These painful paroxysms may occur many times a day and last only a fraction of a second or several seconds, with refractory periods of no pain in between in about 50% of patients. The other half of TN patients will experience continued pain of lower intensity in the same distribution (9). The sudden paroxysmal pain can be elicited by things as simple as light touch of the affected area, wind on the affected area, talking or chewing, and kissing.

TN is classified as in the following three categories

1. *Idiopathic TN*: no apparent cause
2. *Classical TN*: caused by vascular compression of one or more of the trigeminal roots
3. *Secondary TN*: caused by neurological disease such as MS plaque or tumor compression at the cerebellar pontine angle.

TN more often affects the right side of the face, is unilateral, and involves the V3 distribution of the lower face, jaw and tongue (10). Bilateral involvement, which need not be concurrent, or even temporally linked, should prompt strong consideration of MS as the most likely underlying diagnosis.

TN is 20–400 times more common in the MS population than in the general population (11). TN in MS is usually attributed to a plaque in the root entry zone of the trigeminal nerve, although deeper lesions affecting the trigeminal system (especially the trigeminal tract and nucleus) have also been associated with this syndrome.

Conventional treatment is aimed at

1. decreasing neuronal excitability and ephaptic transmissions with so-called membrane stabilizing agents [carbamazepine (Tegretol), oxycarbazepine (Trileptal), phenytoin (Dilantin), gabapentin (Neurontin) valproate (Depakote]
2. analgesia (opiates, nonsteroidal anti-inflammatory drugs)
3. long-acting prostaglandins such as misoprostol (12)
4. anti-inflammatories
5. surgical destruction of nociceptive pathways
6. surgical decompression of ectatic vessels making contact with trigeminal roots (6, 12)

Treatment is usually started with carbamazepine or other sodium-channel blockers, which attenuates or can even abolish these exuberant ephaptic transmissions. The medications utilized should be titrated up until pain relief is achieved or until side effects (confusion, fatigue, ataxia) preclude further dose adjustment. If ineffective, this should prompt a different strategy.

If pharmacologically derived treatments (such as monotherapy or multidrug therapy) fail, patients are generally referred to neurosurgery to discuss gamma knife, rhizotomy or vascular decompression (12). In the authors' experience, other treatment alternatives include “dry needling” of the face and application of analgesic sprays (both on the face and inside of the mouth); recently, we have treated a number of medication-resistant patients with low-dose aripiprazole at 1–2 mg in the am or in the am and pm (personal observations) with surprising efficacy (13).

## “Lancinating My Throat With a Red-Hot Poker; Then into and Out of My Ear”: Pain Associated With Glossopharyngeal and Glossopharyngeal-Vagal Neuralgia

Julie was a 25-year-old woman with a history of RRMS, treated with teriflunomide at 7 mg orally once daily (as treatment at 14 mg daily was associated with significant hair loss). At age 21, she developed right eye pain consistent with right retro-bulbar optic neuritis, which was diagnosed on the day of symptom onset; treatment started the next day utilizing high-dose oral dexamethasone at 80 mg PO with breakfast and lunch daily for 5 days without taper.

Julie's vision recovered over 2 weeks to 20/20 OU, with full visual fields, albeit with a persistent right relative pupillary defect (RAPD). Mild right optic disc pallor was noted 3 months later, principally in the temporal quadrant.

Imaging of the brain and spinal cord revealed an enhancing short-segment lesion in the right optic nerve, best appreciated on coronal T1 fat-suppressed images with gadolinium infusion. Two additional non-enhancing lesions were identified in the deep white matter; both exhibiting an ovoid appearance on axial fluid-attenuated inversion recovery (FLAIR) sequences, and Dawson's fingers on sagittal FLAIR. An additional lesion was identified in the dorsomedial pontine tegmentum, in the region of the medial longitudinal fasciculus (MLF) on the left. Further, a second exam carefully assessed for any evidence of ocular dysmotility, and a subtle but definite internuclear ophthalmoparesis (INO) was identified on the left (i.e., slow adduction of the left eye during saccades to the right). MRI of the spinal cord was normal.

Given breakthrough MS activity, Julie was then slowly escalated to 14 mg of teriflunomide daily. Julie was counseled by a physician assistant (PA) who suggested that Julie first attempt to address limiting hair loss that she previously experienced when escalation of the 14 mg dose was attempted.

This second time it was suggested that Julie start spironolactone (a mild diuretic and utilized for mild cases of acne) given that many patients in the clinic where Julie received her MS management had been using this drug for hair loss associated with interferons, teriflunomide, among others, and on the mechanistic basis of telogen fluvium (14–18). She began with 50 mg daily of spironolactone for 1 week, after which she escalated to 100 mg daily thereafter. If a patient is still losing hair excessively following the escalation to 14 mg of teriflunomide, then another recommendation would be to add topical minoxidil in addition to a multi-mineral product (containing biotin, zinc, selenium, among others). However, she tolerated the 14mg dose of teriflunomide, and conspicuously Julie's hair in fact got thicker. She did well demonstrating no clinical change or radiologic evidence of new activity on MRI at 6 months and then yearly.

On her 25th birthday Julie was out with friends dancing. She went to bed at 5 AM and awakened at 2 PM with what she thought was a sore throat, albeit only localized to the right side. Julie recognized that the pain was abrupt, short-lived, recurrent and localized to the region of the tonsillar pillar, the right posterior aspect of her tongue, and to the posterior pharyngeal wall.

As she loudly yelped with each pulse of severe pain, rated 10 out of 10, and incapacitating, it woke Julie's boyfriend, an emergency room physician, who examined Julie's throat but found no evidence of pathology. While the oscillating pain resolved, it could be triggered by swallowing liquids or food, and sometimes just saliva. Other triggers could include coughing, yawning, talking or even kissing.

The following day, Julie was applying make-up to get ready for work. Her boyfriend was leaving to begin his emergency room shift when he went to kiss Julie goodbye, at which point she screamed from the throat pain, this time radiating into right ear.

He laid her down on the floor with her legs elevated, and Julie awakened within 5 to 10 seconds, which is most consistent with syncope. Upon awakening she described pain akin to “someone lancinating me with a red-hot poker into the right side of my throat, but then changing direction and pushing that poker right into and out of my right ear.”

At this point Julie's boyfriend insisted that Julie accompany him to the hospital and be seen in consultation by neurology. The boyfriend pleaded that the neurologist take a look at Julie.

So that Julie did not need to speak, another trigger of her throat pain, the boyfriend provided a history of Julie's MS and the new syndrome of throat pain, and its singular episode associated with syncope this morning. After further history and a complete general and neurologic examination; leaving the throat for last, the neurologist concluded, “Julie and Rex this is most likely a classic syndrome of glossopharyngeal neuralgia (GN) and glossopharyngeal-vagal neuralgia (GVN) (see Table 2) (19–21). This pain syndrome can be caused by a broad diversity of mechanisms and processes, including secondary to MS. If in fact MS is the culprit, usually this particular pain syndrome is related to a plaque localized to the region of the IX and X cranial nerves (glossopharyngeal and vagus, respectively) in the lowest third of the brain stem, the medulla oblongata.

### Pathophysiologic Mechanisms for GN

The mechanism for both GN and GVN is believed to be electrical discharges described as ephaptic discharges, which initiate in cranial nerve IX and then project to include the vagus, or cranial nerve X. The glossopharyngeal cranial nerve provides sensation to the throat including taste from the posterior 1/3 of the tongue. It also provides sensation to the region of the tonsillar fossa, base of the tongue, the posterior pharyngeal wall, the ear canal, and the tympanic membrane. “Julie, your throat pain radiating to the ear is likely based on this anatomic arrangement. Finally, your fainting suggests electrically exuberant triggering of the vagus, which can lead to bradycardia followed by fainting,” the neurologist opined.

### Application of Local Anesthetic to Mitigate or Abort GN

The neurologist suggested, “Let's do a few things for Julie. First, I'm going to ask for that bottle of Cetacaine with the long applicator attached. We'll spray this composite analgesic of three esters (benzocaine 14%, butamben 2%, tetracaine HCl 2%) into the throat, which often mitigates if not abolishes the pain- at least for a period sufficient for us to image the brain and brain stem to confirm our hypothesis.”

### Differential Diagnosis of GN

We think this is a new MS plaque but there are other causes such as mass lesions and vascular compression syndromes related to enlarged ectatic blood vessels that can compress cranial nerves leading to electrically exuberant syndromes (in GN, the vessels typically implicated in such compression are the posterior-inferior cerebellar artery, a branch of the vertebral artery, and the anterior-inferior cerebellar artery, a branch of the basilar artery). The diagnostic studies of choice include MRI, MR angiography, and 3D computerized tomogrsaphy (CT)-angiography (22, 23).

In this case we must also consider a vessel compression syndrome as well as cerebellar-pontine angle mass lesions such as meningiomas, a glomus tumor, and sarcoid.

### Clinical Semiology of Julie's GN

The topical anesthetic spray abolished Julie's pain. MRI of the brain revealed a new gadolinium enhancing plaque on the right medulla in the region of cranial nerves IX and X. No other abnormalities were identified, including no mass lesions. The neurologist reviewed the images with Julie and Rex and recommended IVMP at 1 g daily for four days and follow up imaging a few weeks later. Further, the neurologist recommended a transition in Julie's MS disease modifying therapy from teriflunomide to natalizumab.

Following the first infusion of IVMP, Julie's pain resolved without further episodes of syncope. The MRI 2 weeks later revealed resolution of the gadolinium enhancing lesion, albeit a new T2/FLAIR lesion was persistent in the right medulla as originally identified.

Julie made the transition to natalizumab, experienced improved energy, and was without clinical or radiologic activity until age 28 (3 years later) when the GN returned. Repeat imaging showed no interval changes and once again Julie and Rex consulted the neurologist. “Well now it appears that we have a chronic pain syndrome ofGN. Sometimes treating early at the time of lesion development will resolve the problem for good. Sometimes not. The good news is that we have a number of medications that can control these unwanted bursts of electricity in the system of cranial nerve IX,” explained the neurologist. These include anti-convulsant agents, gabapentin and pregabalin (known as gabapentinoids), tricyclic antidepressants, as well as aripiprazole which we have found (personal observations) to be extremely effective for cranial neuralgias as well as for neuropathic pain syndromes in general (see Table 2) (19). Aripiprazole has been shown to have anti-nocioceptive effects by a number of different mechanisms (24, 25). Topical anesthetic sprays for the throat (e.g., Cetacaine) can be effective in mitigating or even abolishing GN associated pain. In those who are in a pain cycle recalcitrant to these medications, including escalation of their dosages, then other considerations include pulse high-dose corticosteroids (250–1,000mg of IVMP; 250–1250mg of prednisone) or dexamethasone (40-160mg taken orally in divided doses, or by IV at 160 mg; the latter dose being equivalent to 1 gm of methylprednisolone). Alternately, when patients remain refractory to medication interventions, then we request neurosurgical consultation to consider one of a number of procedures that can be mitigating, and sometimes curative.

Microvascular decompression is a two-step procedure, similar to the Janetta procedure performed for medication resistant TN (26), whereby 76% of those undergoing this procedure exhibit improvement (27). The steps include vascular decompression (if a vessel ectasia is in fact identified) whereby vessel contact with the nerve root can lead to segmental demyelination. The vessel is dissociated from the nerve root, with a pledget (i.e., a pad) placed in between the vessel and nerve root, in order to subsequently prevent further contact. Also, a second step involves a delicate and high precision scrape and cut of the sensory component of the nerve root (which may be performed in isolation when a vascular compression is not identified intraoperatively, as when the syndrome is caused by an MS plaque). Other procedures include rhizotomy, nerve block, pulsed radiofrequency neurolysis, gamma knife, and stereotactic radiosurgery (28, 29).

Julie was started on pregabalin titrating up to 200 mg twice daily with excellent control until 3 months later when she experienced breakthrough. Higher doses of pregabalin were not tolerated secondary to sedation, hence a small dose of aripiprazole was recommended at 2 mg in the morning, which abolished the pain completely. A year later Julie remains pain-free on pregabalin and aripiprazole, and in MS remission with improved energy and quality of life.

## “My Leg is Stiffer Than My Old Law School Professor and A Bigger Pain Too”: Pain Associated With MS Spasticity

Denny is a lawyer who noted difficulty with his right leg when jogging for the past 18 months. Initially, he only noticed some stubbing of his toe at the end of his run, but recently he noted his leg is getting stiff and sometimes will drag at the end of the day. In addition to his stiffness, he also noted occasional painful spasms in his right lower extremity.

His past medical history was only significant for well-controlled hypertension, which he managed with lisinopril.

On exam, he had bilateral weakness in both iliopsoas and tibialis anterior muscles, which was worse on the right. He had increased tone and reflexes in the right leg with an upgoing plantar response. Vibratory sensation was decreased in both great toes under 10 s. Visual acuity was also slightly decreased in the left eye.

Routine blood work was unremarkable. Vitamin B12 level was normal. Lyme antibody titer was negative. Vitamin D level was just below normal limits.

MRI of the spinal cord showed two nonenhancing lesions, one at C4 and the other at T10.

MRI of the brain showed four nonenhancing lesions, located in periventricular and juxtacortical locations and one enhancing periventricular lesion.

Cerebrospinal fluid (CSF) was positive for oligoclonal bands and IgG index was 0.53. Cell count and total protein were normal.

Denny met criteria for primary progressive multiple sclerosis (PPMS). Most clinical trials were negative for PPMS until the ocrelizumab trial (30). The rituximab trial had shown some promising results in patients who were younger or who had active disease suggesting that these patients were the ones that most likely benefited from treatment (31).

Denny was started on ocrelizumab to help slow progression of his disease, due to his young age and because he had a fairly active life style. It was also pretty clear that the symptom limiting his activity was his spasticity (a velocity-dependent increase in muscle tone). The painful spasms were also bothering him. Denny was initially prescribed baclofen, but he felt the medication made his sleepy and he did not like that it affected his ability to be sharp in his work as an attorney.

Spasticity is a common symptom in MS due to demyelinating lesions in the spinal cord and brainstem. Spasticity produces stiffness and fatigability of the muscles as well as spasms, painful cramps, and clonus (32). Although few studies on the mechanism of action have been done, clinically, spasticity seems to be composed of two types: phasic spasticity, which begins with spasms, painful cramps, and clonus, and tonic spasticity, which produces stiffness. This spasticity can limit ambulation, impair balance, increase the risk of falls, produce pain, increase exertional fatigue, and interrupt sleep (32). Historically, baclofen, dantrolene, benzodiazepines, and tizanidine have been used to treat painful spasms (see Table 2). Although proven efficacious, these medications are associated with a high incidence of sedation or weakness and may produce untoward effects on cognition and balance in patients with MS. For some patients, some of these side effects can be improved with the administration of intrathecal baclofen, which provides baclofen right where it is needed most in the spinal cord with little effect on the brain itself (33). Hepatotoxicity is also a well-recognized complication of dantrolene, and abnormal liver test results have been reported with both tizanidine and baclofen therapy (34).

Some studies indicate that an antiepileptic drug, gabapentin, may be effective in treating spasticity (35). In our clinical experience, the drug seems to have greater efficacy for cramps and spasms (phasic spasticity) than for the stiffness associated with spasticity (tonic spasticity), although an effect on both types of spasticity has been noted in the published literature (36). The precise mechanism responsible for the effectiveness of gabapentin is not known.

Levetiracetam is a second-generation antiepileptic drug used as treatment for partial-onset epilepsy. Its mechanism of action has not been fully elucidated, but it is known to promote inhibitory neurotransmission via indirect modulation of gamma-aminobutyric acid_A_ and glycine receptors (37) and to depress high-voltage–activated calcium currents by inhibiting N-type calcium channels (38). Levetiracetam has shown efficacy in the treatment of neuropathic pain in humans and in an animal model of neuropathy (39).

Because of the pain and tonic spasms experienced by Denny, he was switched from baclofen to an anti-epileptic agent known to have few cognitive side effects. He was started on a dose of 250 mg of levetiracetam daily that, over 4 weeks, was increased to a dose of 1,000 mg twice a day. The patient noted significant improvement in the painful tonic spasms but still had some increased tone in his right lower extremity. Overall, he was pleased that his symptoms had improved with little in the way of adverse side effects.

## “Did I Get Punched in the Eye?”: Pain Associated With Optic Neuritis

Jessica was a 34-year-old accountant with no significant past medical history who presented to her internist's office with 3 days of left eye pain, which was worse with eye movement. She noted blurry vision beginning that morning. She was referred to an ophthalmologist, who reported a normal examination except for decreased visual acuity of the left eye, an afferent left pupillary defect, and decreased color vision in the left eye. Funduscopic examination was normal.

She was referred to a neurologist who conducted an in-depth history and physical examination. A more detailed history illuminated an episode of 10 days of numbness in the right foot 3 years earlier. At that time, she was about to schedule a doctor's appointment when the numbness resolved, and she did not think much of it. Her neurologist ordered a battery of laboratory tests, including antinuclear antibody (ANA), rapid plasma regain (RPR), vitamin B12, folate, and rheumatoid factor (RF), all of which returned negative results.

The CSF analysis revealed the following:

- 60 mg/dL CSF Glucose
- 30 mg/dL Protein
- 5 per mL White Blood Cells
- 1 per mL red Blood Cells
- 3.5 IgG Index (Elevated)
- 4 Oligoclonal Bands

MRI of the brain showed two periventricular lesions, three juxtacortical lesions on axial FLAIR imaging. Post gadolinium axial T1 imaging showed an enhancing lesion in the left optic nerve.

She was given 3 days of pulse steroids with a taper; her vision improved, and her pain diminished significantly.

Patients with optic neuritis associated with MS present with subacute loss of vision that develops over a period of hours or days. In about 90% of patients, the vision loss is associated with orbital pain that is worse with eye movements (40). The pain is thought to be due to irritation of the inflamed optic nerve, particularly when the nerve gets stretched with eye movement (41). Endoneural inflammation is also thought to activate intraneural pain receptors in optic neuritis. The character of the pain in optic neuritis is typically of a dull character and not the lancinating, electrical pain often associated with neuropathic pain.

While high dose corticosteroids do not typically have long term benefits in MS patients with acute optic neuritis, they are usually given to patients, as in this case, because they help with resolution of pain and usually speed up resolution of inflammation and visual recovery (40). Because the pain and loss of vision are worrisome for most patients, they tend to see a physician early in the disease course.

Painful optic neuritis is not only a manifestation of MS, but of other demyelinating disorders as well (see Table 3). Myelin oligodendrocyte glycoprotein antibody-associated disease (MOGAD) is a disorder in which patients have detectable antibodies to MOG. Because the optic nerve has oligodendrocytes that highly express MOG, it is not surprising that optic neuritis occurs frequently with this disorder (42). In one study, 5/8 patients with MOGAD had painful optic neuritis (43). CSF cell counts were not significantly different between MOG-positive vs. MOG-negative patients. MOGAD patients typically are negative for oligoclonal bands, which differs from typical patients with MS (43, 44). Another difference between MS and MOGAD in terms of their clinical presentation is that optic disc edema is present in a high percentage (86%) of patients with MOGAD in one study, while rarely present in MS, who typically have retrobulbar neuritis (44). Patients with optic neuritis due to MOGAD recover quite well after a course of steroids, including recovery from their pain.

**Table 3:** Comparison of features of demyelinating syndromes.

**Characteristic**	**Multiple sclerosis**	**NMOSD**	**MOGAD**	**Idiopathic TM/ON**
Bilateral ON	Rare	Often	Often (CRION)	No
Lesions other than brain and spinal cord	Yes. Periventricular white matter and juxtacortical areas common	Hypothalamus, area postrema, periaqueductal gray matter		No
Oligoclonal bands	Almost always	Rarely	Never	Rarely
Longitudinally extensive transverse myelitis	Rare	Common	Common	Rare
Relapses	Yes	Yes, often severe	Rare	No
CSF Protein	Normal	Increased	Increased	Can be increased
Specific test for disorder	No	AQP4 antibody	MOG antibody	No

*AQP4, aquaporin-4; CRION, chronic relapsing inflammatory optic neuropathy; CSF, cerebrospinal fluid; MOG, myelin oligodendrocyte glycoprotein; NMOSD, neuromyelitis optica spectrum disorder; ON, optic neuritis; TM, transverse myelitis*.

## “When I Look Down, Boy Do I Have a Pain in the Neck”: Pain Associated With Lhermitte's Phenomenon

Ruth was a 44-year-old African American woman with a past medical history depression and migraines. At time of presentation, she was having some excessive fatigue and then noted blurred vision in her left eye. She was diagnosed with RRMS after an episode of optic neuritis as her first presenting symptom, but MRI of the brain showed three other enhancing lesions as well as several nonenhancing periventricular lesions. Diagnosis was based on the 2010 McDonald criteria for Multiple Sclerosis. At time of diagnosis, she was given 1 gram IVMP for 3 days and completed a prednisone taper over the course of 2 weeks. One year later, she began to note some tingling in both upper extremities. This improved over a few weeks, but she then developed a painful, electrical-like sensation radiating into both arms, particularly with neck flexion. MRI of the cervical spine showed a nonenhancing lesion at the level of C5-6.

Lhermitte's phenomenon is defined as “a transient, brief sensation related to flexion of the neck and felt in the back of the neck, lower back, or in other parts of the body” (2). Although not specific for MS, Lhermitte's phenomenon is frequently associated with MS (45). In many patients, this symptom is transient, and manifests only for some weeks, then resolves spontaneously (46). The reported prevalence of Lhermitte's phenomenon varies widely. The three studies that analyzed Lhermitte's phenomenon in at least 100 patients with MS, found it in 313 patients out of 2,085 patients, yielding an overall prevalence of 15 % (47). Lhermitte's phenomenon probably depends on a demyelinating plaque in the dorsal columns at the cervical level, as suggested by two MRI studies that found a strong correlation between Lhermitte's phenomenon and the presence of MS plaques in the posterior cervical spine (48). It should be noted that other conditions, including common conditions such as cervical disc degeneration can also cause Lhermitte's phenomenon.

Although Lhermitte's phenomenon is a frequent sensory disturbance in patients with MS, few patients report this symptom spontaneously and only few consider it painful. A clinical trial examined the use of injected lidocaine and oral mexiletine for patients that had painful paroxysms (49). Injections of lidocaine achieving a plasma level of 2.4 pg/ml are often helpful in treating MS pain, suggesting that the pain mechanism is mediated through sodium channels. Oral mexiletine (300–400 mg/day), a derivative of lidocaine, obtained similar results. Sodium channel blockers such as carbamazepine, lamotrigine and phenytoin have also been used to treat painful Lhermitte's phenomenon, again suggesting that these channels are involved in mediating pain in certain types of MS lesions (see Table 2).

Pain may also be exacerbated in Lhermitte's phenomenon because of the presence of tumor necrosis factor-alpha (TNF-alpha) in the lesion (50). TNF-alpha has been shown to increase impulse activity in unmyelinated Ad and C fibers, which mediate nocioception (51). This effect may be mediated by low concentrations of TNF-alpha since higher concentrations were noted to reduce firing of these nerves.

Patients with other demyelinating disorders can also experience painful myelitis (see Table 3). For example, patients with neuromyelitis spectrum disorder (NMOSD) often have longitudinally extensive transverse myelitis, and these patients are more likely to have higher scores on the pain effects scale (PES) than MS patients with typical transverse myelitis (52). NMOSD can cause severe, persistent pain, which is most often central neuropathic pain (53). This pain is described as burning, shooting or lancinating in character. It is thought that the pain results from changes due to reorganization of the somatosensory system in the spinal cord (54). Because the damage from transverse myelitis in NMOSD often leads to tissue necrosis, disruption of sensory pain tracts occurs, leading to severe pain in these patients (55). Thus, transverse myelitis in NMOSD is frequently associated with severe pain that is often poorly controlled with pharmacologic interventions (55).

Lesions in NMOSD are typically in the cervical and thoracic cord and are often longitudinally extensive (greater than three spinal segments). The target of the antibodies in NMOSD is the aquaporin-4 (AQP4) channel that is mostly expressed on astrocytes (42). Pain appears to be more severe in lesions that are large enough to involve the spinothalamic tracts (56). There is also high density of AQP4 in the brainstem, including the trigeminal nucleus and the periaqueductal gray matter. Because of involvement of these areas, neuropathic facial pain and headache can also result in patients with NMOSD (42).

Central neuropathic pain can be treated with gabapentin, pregabalin, or levetiracetam (57, 58). Trials utilizing duloxetine have also shown benefit in central neuropathic pain caused by spinal cord injury such as that occurring in NMOSD (59).

In addition to NMOSD, MOGAD can also be associated with severe, debilitating pain (42). Unlike the target in NMOSD, MOG is predominantly expressed on oligodendrocytes, but spinal cord lesions can also affect the spinothalamic tracts. In addition to spinal cord lesions, brainstem lesions also occur, often leading to TN and headache (42). While spinal cord lesions can also be longitudinally extensive, the lesions often cause sensory symptoms, including painful dysesthesias.

Similar to NMOSD, initial treatment of MOGAD is typically IVMP, 1,000 mg for 3–5 days. If the pain still exists after the initial anti-inflammatory treatment, tricyclic antidepressants (e.g., nortriptyline and amitriptyline), serotonin and norepinephrine uptake inhibitors (e.g., duloxetine and venlafaxine), or gabapentanoids (e.g., gabapentin or pregabalin) are used to treat the pain (42) (see Table 2).

## “Orgasm Transformation into A Volcanic Eruption of Exploding Electricity”: Pain Associated With Pathophysiologic Reorganization of ‘G-Spot' Activation Secondary to MS Myelitis

Taylor was a 27-year-old woman who got married last year to her high school sweetheart Bill. Two months ago, Taylor developed a sensory disturbance localized to her lower abdomen extending to her rectal and vaginal region and characterized by newer anesthesia. She had minimal sensation to passing urine and stool and no sensation during arousal or intercourse. She urgently was evaluated in neurologic consultation, at which time the examination revealed diminished sensation to soft touch, pinprick sensation, and cold stimulus from the umbilicus to the inferior buttocks as well as the saddle including the anus and vaginal regions.

Imaging revealed an enhancing lesion from about T-10 to the conus medullaris without root involvement. Brain imaging revealed disseminated lesions including ovoids and Dawson's fingers, while examination revealed right optic disc pallor and a right RAPD. Further, the patient had subtle but definite gaze-evoked nystagmus, with rebound nystagmus upon return to center, and saccadic pursuit eye movements; all of these were consistent with involvement of the neural integrator, most prominently the cerebellar flocculus.

MS mimics were excluded and CSF demonstrated seven unique oligoclonal bands with an elevated IgG index of 2.2; blood studies were negative for AQP4 and MOG antibodies.

Taylor was diagnosed with MS and treated with 3 days of IVMP without improvement. She was then advanced to plasma exchange at one volume daily for five total volume exchanges over 8 days to allow for correction to fibrinogen. With plasma exchange, she completely recovered with normalization of her sensations in the affected areas. She was started on natalizumab at every 8 weeks in lieu of being John Cunningham virus (JCV) IgG positive.

Taylor did very well with improved energy and mood until 3 months later, when she began to experience a highly unusual sensation during intercourse. This was characterized as the perception of sliding ground glass when her husband's penis made contact with her ventral vaginal vault, typically inducing orgasm (mediated by the so-called Grafenberg or G-spot) (60, 61); however, now it instead triggered an electric lightning-bolt of radiating pain into the vaginal region and rectum. Dyspareunia is characterized by pain during intercourse in general, whereas the pattern experienced by Taylor is an electrical (ephaptic) neuropathic variant of dyspareunia.

During these episodes of painful intercourse Taylor simply cried but did not communicate with her husband about the pain which provoked her tears. Bill believed that Taylor was happy. However, when visiting with her and Bill in clinic, and moving through the review of systems, upon asking about sexual issues and symptoms of dysfunction Taylor began to cry. Her husband admitted that “she also cries during sex; I thought she was happy.” The review of her symptoms prompted Taylor to finally open up and describe the torture she was experiencing. It was like an “orgasm transformation into a volcanic eruption of exploding electricity…like nothing I have ever experienced before.”

### Post-myelitis Neuropathic and Transformed Pain Syndromes

We reviewed the post myelitis neuropathic pain syndrome, whereby the lesion localized to the region of afferent input from “G-spot” activation, becomes reorganized in such a way that rather than triggering orgasm, the affected region of the spinal cord instead mistakenly responds with an uncoordinated cluster of ephaptic electrical discharges in a neuroanatomically disorganized pattern. These discharges are now perceived as radiating volleys of pain into the vaginal and rectal regions.

Taylor was re-examined to ensure that there were no new deficits; and there were none. Repeat imaging showed the old lesion from the prior study but no enhancements or other changes. We then discussed how chronic pain can become transformed with new synapse formation, transforming local circuit and network activity, forming the pathophysiologic basis for pain syndromes that, if not interrupted or modified, can become hardwired and even treatment resistant.

We discussed a variety of medication options, but the systemic side effects were not agreeable with Taylor, especially with her work as a pediatric neuro-rehabilitation specialist. As such, we recommended that she try belladonna (15 mg) and opium (30 mg) (B&O) supprettes, a formulation long-used in patients following colorectal surgery in order to mitigate regional pain syndromes in a site-selective-delivery strategy (62). The active components in B&O supprettes are absorbed by the inferior and superior rectal veins producing pelvic analgesia within 20–30 min in most (63, 64).

In our MS and Neuroimmunology practice, we have also utilized this highly effective strategy in our patients with pelvic and genitourinary pain syndromes; particularly those associated with pain sequelae following inflammatory myelitis.

We educated Taylor and Bill about B&O supprettes, and advised inserting one per-rectum, 1 h before sexual intercourse. The result was complete abolishment of the pain syndrome. I suggested that Taylor reduce the dose by 1/4 suppress each month until off to determine if we could demonstrate the reversal of this *transformed pain syndrome*. It worked and she has remained off the medication for 1 year without recurrence. Her MS was under excellent control with extended interval dosing at 8 weeks of natalizumab.

## “At Least in My Case, It's Like the Treatment is Worse Than the Disease!” Disease Modifying Therapy-Associated Pain Syndrome: Mechanisms and Management

Wendy was a 45-year-old woman with a history of RRMS, characterized by right retrobulbar optic neuritis at age 25, right hand and arm numbness in a circumferential distribution in conjunction with painful electric and vibratory pain with neck flexion (i.e., Lhermitte's) and in association with an enhancing MS plaque at C5-C6 at age 28. She experienced an episode of myelitis at age 44, producing sensory and motor manifestations affecting both legs in addition to diminished sensation to passing urine and stool, as well as during intercourse. Imaging revealed an enhancing plaque from about T12 to the conus medullaris ~L1. Each of these exacerbations resolved completely with IVMP at 1 gm/day for 3 days without taper for the first two, and with oral dexamethasone at 80 mg with breakfast and lunch for 5 days without taper.

Wendy was treated initially with weekly intramuscular interferon beta-1a, until her second exacerbation, at which time she was converted to high-dose interferon beta-1a at 44 mcg taken subcutaneously three times weekly. Surveillance studies revealed a high interferon neutralizing antibody titer 1 year later at 1:1,024, prompting transition to daily subcutaneous glatiramer acetate, which she utilized adherently for 7 years until early 2013. She developed injection fatigue and extensive lipoatrophy, which prompted conversion to oral teriflunomide at 14 mg daily. Last year, she experienced severe sensory and motor myelitis despite adherent use of teriflunomide, which prompted conversion to oral dimethylfumarate at 240 mg taken twice daily.

Despite initially taking 120 mg twice daily, and escalating to 240 mg twice daily one month later, Wendy described facial flushing with each dose along with loud and embarrassing bowel sounds (i.e., borborygmi), frequent watery diarrhea preceded by extremely painful tenesmus, in addition to abdominal pain occurring in isolation throughout the day. Further questioning revealed that each of the symptom episodes also was attended by some mild, albeit noticeable, lacrimation and salivation. “At least in my case, it's like the treatment is worse than the disease!” she noted.

In order to address Wendy's symptoms with dimethylfumarate we had her take baby aspirin at 81 mg just prior to each of her two daily doses, which abolished her facial flushing, but did not affect her GI symptoms, lacrimation, or salivation. As such we then added montelukast at 10 mg, taken along with the baby aspirin, reducing her diarrhea, but not the abdominal pain, lacrimation or salivation. Noting that such symptoms in combination were reminiscent of those we have observed in myasthenia gravis patients taking pyridostigmine, and which can be markedly mitigated, if not abolished, with pre-treatment using a muscarinic anti-cholinergic agent (so as to not interfere with the effects of pyridostigmine at the nicotinic-receptor-mediated neuromuscular junction function) such as glycopyrrolate, we decided to add 1 mg of this agent, to be taken together with the baby aspirin and montelukast about 5–10 min prior to each of the dimethylfumarate doses. This combination of pre-treatment completely abolished all of Wendy's symptoms associated with each of the two daily doses of dimethylfumarate.

Given the complexity and twice daily dosing regimen, it was decided to transition Wendy to once daily diroximel fumarate 231 mg time-release capsule. Upon treatment inception, without pre-treatment, Wendy experienced facial flushing and a more protracted warm sensation throughout, when compared to her prior regimen of twice daily dosing of dimethlyfumarate, which produced reversible changes in the vision of her right eye (consistent with Uhthoff's phenomena, which she has also noted following a hot shower or bath). Further, while her diarrhea was markedly reduced, she still complained of abdominal pain in conjunction with mild lacrimation and salivation. It was decided to not resume the montelukast, but to instead simply add back the glycopyrrolate at 1 mg, 5 min before dosing of the diroximel fumarate, in conjunction with the baby aspirin. It worked and all of her dosing symptoms were resolved, with the added benefit of only once daily dosing.

## “A Couple Should Not Be so Close, as to Become Inseparable; Ouch!!!” Pain Associated With Arousal-Triggered Vaginismus and Intercourse-Associated Penis-Captivus

Maggie was a 60-year-old woman with secondary progressive SPMS, whose course is characterized by bilateral internuclear ophthalmoparesis (INO) at age 21 during her senior year in college. During her final exams, she experienced abnormal horizontal eye movements, producing transient ‘ghosting diplopia' and oscillopsia. Pulse oral prednisone over 1 week produced marked improvements, but not without residual deficits; especially when exposure to high ambient temperatures (e.g., in the summer, after a hot shower or bath, or after a fight with her husband about finances; all consistent with Uhthoff's phenomena). No disease modifying therapy was offered at that time.

At age 23, she developed dysarthria, ataxia, and right arm tremor during her second-year in law school. She was evaluated in the hospital, diagnosed with RRMS, treated with 5 days of IVMP, and then treated with azathioprine and dose escalation until the lymphocyte count was ~800 and the red cell mean corpuscle volume (MCV) was >101. She remained in remission (clinically and radiographically) until age 35 when she presented with partial sensory myelitis affecting her right side with numbness and paresthesias from the upper thigh to the axilla. She was transitioned to high dose interferon beta-1b taken subcutaneously three times weekly and did well, with normal surveillance labs and no evidence of an interferon neutralizing antibody assessed yearly. However, at age 40, Maggie awoke one morning with right leg weakness, followed 3 days later by left leg weakness. An examination revealed severe paraparesis (rated at 3- throughout the lower extremities), brisk deep tendon reflexes throughout, both toes upgoing, and a sensory level at T-10. Post-void residual urine volume was 350cc, requiring temporary catheter placement. MRI revealed a new enhancing plaque from about T9 to T11, with varied geometry and affecting the lateral columns on the left and right at different levels; MRI also demonstrated a lesion in the dorsal column at about T9.

Maggie was admitted for plasma exchange 1 volume for a total of 5 volume plasma exchanges, punctuated by the administration of IVMP at 1 gm directly following each of the plasma exchanges. Maggie recovered rapidly, spent a brief 5 days in neurorehabilitation for gait assessment and to establish a bladder program. She was then transitioned to oral mycophenolate mofetil titrating to 1 gm twice daily on an empty stomach in conjunction with pulse IVMP given as 1 gm for 1 day monthly.

Maggie did remarkably well until age 55, at which time she began to exhibit features of SPMS, with a slow course of motor decline, requiring the use of a cane at age 56; and a walker by age 58. Further, she began to experience nocturnal flexor and extensor spasms in her legs which were markedly improved with clonazepam at 0.5 mg taken at bedtime, and with a brief stretching program right before bedtime.

Maggie presented at age 60 with a highly unusual complaint. Specifically, over the course of a week, and during intercourse, Maggie had developed a highly unusual painful spasm localized to her vaginal region, which she perceives to be at the entrance where her husband inserts his penis (i.e., the vaginal introitus), a muscular-spasm variant of dyspareunia. More conspicuous was her husband Brett's addition to the discussion, whereby he stated, “and, I am completely unable to remove my penis, and it's horribly painful. Eventually, and with exhaustion upon trying to remove it, the spasm resolves, and we can separate.” He further added, “I don't believe that a couple should be so close, as to become inseparable; ouch!!!”

While this is an extremely rare event, we suspect that this is an example of vaginismus, characterized by the abrupt burst of ephaptic discharges from the spinal cord to the musculature of the vaginal introitus, and often in conjunction with pelvic floor muscles, culminating in an alarmingly painful and powerful clamping down of the vaginal vault. When this occurs, the inserted penis can become trapped; what is called “penis captivus” (65). To our knowledge, this is the first report of vaginismus-associated penis captivus in MS.

Upon further discussion, it was clear that despite my review of a variety of medications capable of potentially abolishing such activity, Maggie was not keen upon taking anything systemically that might compromise her already marginal balance. Escalating the clonazepam was something she already tried on her own up to a total dose of 2 mg without benefit.

We decided to image the spinal cord to ensure that this highly unusual and conspicuous syndrome was not a reflection of new MS spinal cord activity; it was not. Then we suggested, what Maggie and Brett first felt to be highly peculiar. We informed Maggie and Brett that quinine has been well known to have membrane stabilizing effects, and for years was used for spasms and painful cramps, especially those occurring at night and most commonly in the elderly. While taken off the market because of adverse side effects, tonic water contains quinine (the purpose of which is to provide the bitter taste which is characteristic of this cocktail mixer), an amount that while regulated by the FDA, is still sufficient to provide benefits upon cramps and spasms in some patients (66). We instructed Maggie to drink a 7.5 oz can of tonic water 1 h before any sexual activity (important in that Maggie later mentioned that the vaginismus would begin even during the period of arousal prior to intercourse). It worked completely. Over time, she tapered down the 7.5 oz can of tonic, and after about a month was able to resume normal sexual activity without vaginismus or its consequence of penis captivus upon her husband.

## Conclusions

As these clinical vignettes illustrate, pain is a common manifestation of MS and can have a severe impact on a patient's quality of life. In addition to causing an unpleasant sensation or discomfort, pain can affect a patient's emotional well being. It is hoped that by understanding the underlying pathophysiology of these pain syndromes (67), one can provide better management strategies to help patients with MS cope with these manifestations of their disease.

## Author Contributions

EF and TF contributed cases and discussion. MR contributed cases, discussion, and assembled final manuscript. All authors edited final manuscript.

## Conflict of Interest

The authors declare that the research was conducted in the absence of any commercial or financial relationships that could be construed as a potential conflict of interest.

## Publisher's Note

All claims expressed in this article are solely those of the authors and do not necessarily represent those of their affiliated organizations, or those of the publisher, the editors and the reviewers. Any product that may be evaluated in this article, or claim that may be made by its manufacturer, is not guaranteed or endorsed by the publisher.
